# Association between monocyte-to-high-density lipoprotein cholesterol ratio and all-cause mortality in stroke patients: Exploring the potential mediating role of serum creatinine in a NHANES-based study

**DOI:** 10.1097/MD.0000000000045298

**Published:** 2025-10-17

**Authors:** Bo Hei, Jia Ouyang, Jixia Fang, Qun Gao, Bin Wang, Jingru Zhou

**Affiliations:** a Department of Neurosurgery, Peking University People’s Hospital, Beijing, China.

**Keywords:** all-cause mortality, monocyte-to-high-density lipoprotein cholesterol ratio, stroke

## Abstract

Identifying reliable prognostic biomarkers is crucial for the effective management of stroke patients. The monocyte-to-high-density lipoprotein cholesterol ratio (MHR), calculated as the ratio of blood monocyte count to high-density lipoprotein levels, is commonly used to assess the relationship between inflammation and cardiovascular or cerebrovascular health. MHR may play an important role in the prognostic management of stroke. This study aimed to investigate the association between MHR and all-cause mortality in patients with stroke. This was a retrospective observational study using data from the National Health and Nutrition Examination Survey in the United States. A restricted cubic spline analysis was conducted to visualize the relationship between MHR and the risk of all-cause mortality among stroke patients. Weighted Cox proportional hazards models were employed to assess the independent association between MHR and all-cause mortality. Mediation analysis was performed to explore the indirect effect of MHR on mortality through serum creatinine (Cr). A total of 1513 patients were included in the study, of whom 614 died and 899 survived. Restricted cubic spline analysis revealed a positive association between MHR and all-cause mortality in stroke patients. Patients were categorized into a high MHR group (>0.47) and a low MHR group (≤0.47). After adjusting for relevant covariates, the weighted Cox model showed that patients in the high MHR group had a significantly increased risk of all-cause mortality (HR: 1.172, 95% CI: 1.04–1.32, *P* = .009). Stratified and interaction analyses confirmed the stability of the core findings. Mediation analysis indicated that Cr partially mediated the association between MHR and all-cause mortality in stroke patients, accounting for 10.81% of the total effect. Elevated MHR is associated with a higher risk of all-cause mortality in stroke patients, and this relationship is partly mediated by Cr, underscoring the potential importance of renal function in modulating inflammation-related mortality risk.

## 1. Introduction

Stroke ranks as the 2nd most common cause of death worldwide, claiming nearly 7 million lives each year, and stands as the 3rd leading contributor to global disability.^[1–3]^ Early identification of factors influencing stroke prognosis can help prevent premature death in some patients, offering significant benefits for both individuals and clinical practice. However, the current understanding of stroke prognostic factors is still underdeveloped, with few clinically applicable indicators available, most of which lack adequate accuracy.

Emerging evidence suggests that both inflammation and oxidative stress play critical roles in the initiation and progression of stroke. Inflammation plays a critical role in the pathogenesis of atherosclerosis and cerebrovascular diseases, serving as a key contributing factor to various subtypes of ischemic stroke, including large artery atherosclerosis and cardioembolic stroke.^[4–6]^ Shi et al found that elevated SII levels are associated with an increased risk of stroke.^[7]^ Similarly, Yuan et al reported that an increased C-reactive protein to albumin ratio was linked to a higher long-term mortality risk in stroke patients.^[8]^ Likewise, oxidative stress is recognized as a fundamental mechanism underlying various neurological disorders, including stroke.^[9,10]^ Lei et al found that higher levels of the oxidative balance score were associated with a reduced risk of all-cause mortality among stroke survivors.^[11]^ Notably, both high-density lipoprotein (HDL) and monocytes are closely linked to inflammatory and oxidative pathways, highlighting their potential relevance in stroke pathophysiology.

The monocyte-to-HDL-C ratio (MHR) has emerged as a novel, integrative marker that reflects the balance between pro-inflammatory and anti-inflammatory processes. Owing to its practicality and clinical applicability, MHR has been increasingly recognized as a promising indicator for vascular inflammation and has been implicated in the pathophysiology of atherosclerosis, coronary artery disease, and other cardiovascular and cerebrovascular disorders.^[12–15]^ HDL-C is known for its vasculoprotective properties, including antithrombotic, antioxidative, and anti-inflammatory effects,^[16–20]^ partially mediated through the suppression of macrophage recruitment and activation.^[21]^ Conversely, monocytes play a pivotal role in initiating and sustaining inflammatory responses and oxidative injury by infiltrating vascular lesions.^[22]^ As such, MHR offers a composite measure that captures both systemic inflammation and oxidative stress, key mechanisms involved in stroke development.

To the best of our knowledge, no large-scale population-based studies have specifically investigated the association between MHR levels and stroke risk among the U.S. population. Given the growing recognition of MHR as a surrogate marker of systemic inflammation and oxidative stress (both of which are pivotal in stroke pathogenesis) we conducted this study using data from the National Health and Nutrition Examination Survey (NHANES). Our objective was to investigate the correlation between MHR and all-cause mortality in stroke patients through a population-based survey, which reflects the health status of adults across the United States.

## 2. Materials and methods

### 2.1. Data source

This study was a retrospective observational analysis based on data from the NHANES, an ongoing, cross-sectional, nationally representative survey in the United States. As a major program of the National Center for Health Statistics, NHANES is approved and sponsored by the Centers for Disease Control and Prevention and aims to assess the health and nutritional status of the noninstitutionalized civilian U.S. population.^[23]^ The survey is conducted every 2 years using a complex, multistage probability sampling design. Data collection includes in-home interviews, followed by physical examinations at Mobile Examination Centers, where blood and urine samples are also obtained. The NHANES protocol is reviewed and approved by the National Center for Health Statistics Research Ethics Review Board, and written informed consent is obtained from all participants.^[24]^

### 2.2. Study population

Stroke is a global public health issue that affects millions of individuals and imposes a substantial economic burden during the recovery process.^[25]^ Despite significant advancements in the diagnosis and treatment of stroke over the past decades, its incidence continues to rise, particularly with increasing age.^[26,27]^ In recent years, however, the onset age of stroke has shown a downward trend, which is believed to be associated with several modifiable risk factors, such as hypertension, hyperlipidemia, obesity, smoking, and substance abuse.^[28]^

In this study, adults aged 18 years and older were included. Stroke was defined as the dependent variable. Participants were identified as having experienced a stroke if they answered “yes” to the following questionnaire item: “Has a doctor or other health professional ever told you that you had a stroke?”

**Figure 1.**
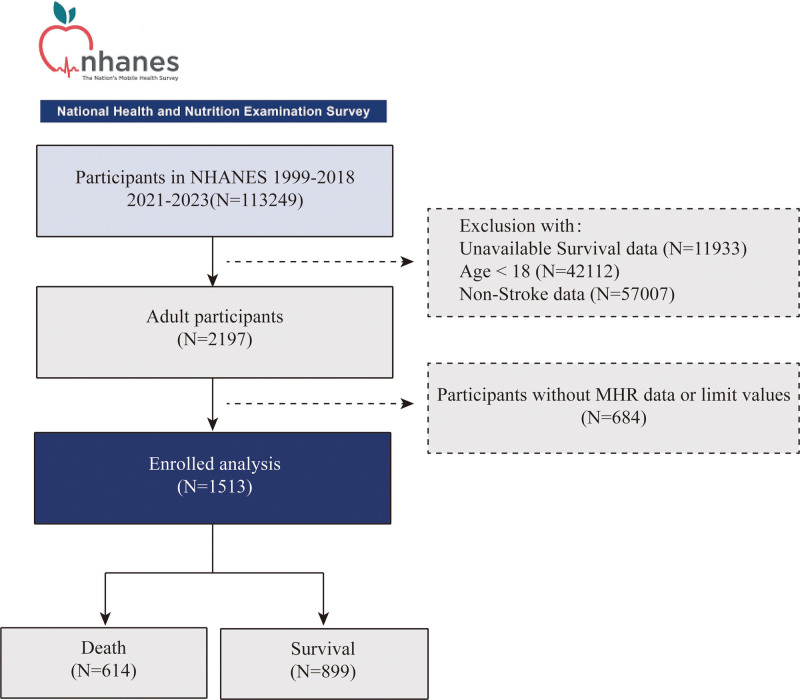
Flowchart of the sample selection from the NHANES. NHANES = National Health and Nutrition Examination Survey.

In this study, we utilized data from the NHANES cycles spanning 1999 to 2018 and 2021 to 2023, which included complete information on stroke status and survival outcomes. The NHANES data from the 2019 to 2020 cycle was excluded due to disruptions in data collection caused by the COVID-19 pandemic, which impacted the completeness and reliability of the dataset during that period. A total of 113,249 participants were initially extracted. Participants were excluded based on the following criteria: missing survival-related data (N = 11,933); age under 18 years (N = 42,112); absence of stroke diagnosis (N = 57,007); and missing or extreme MHR values (MHR > 1.5) (N = 684). After applying these exclusion criteria, a final sample of 1513 participants was included in the analysis, comprising 614 deaths and 899 survivors (see Fig. 1).

### 2.3. Ascertainment of mortality and follow-up

The primary outcome of this study was survival status and corresponding survival time. Mortality status was determined by linking NHANES data to the National Death Index records available at https://www.cdc.gov/nchs/data-linkage/mortality-public.htm. Survival time was calculated from the date of the NHANES examination to the date of death or the end of follow-up, whichever occurred first. Participants who were discharged without a recorded death event were classified as alive at the end of the follow-up period.^[29]^

### 2.4. Assessment of covariates

In this study, several potential confounding factors were analyzed, including age, sex, race, the ratio of family income to poverty (PIR), smoking history, hypertension, body mass index (BMI), cardiovascular disease, urinary albumin, red blood cell count, blood urea nitrogen (BUN), serum creatinine (Cr), lactate dehydrogenase (LDH), and serum albumin. Age, sex, and race were determined using the demographic variables file (DEMO). Participants were categorized into the following racial groups: Mexican American, non-Hispanic Black, non-Hispanic White, other Hispanic, and other races. Based on the family PIR, income levels were categorized as low income (≤1.3), middle income (1.3–3.5), and high income (>3.5). Smoking history, hypertension, and cardiovascular disease were assessed via questionnaire data. Laboratory indicators, including urinary albumin, red blood cell count, BUN, Cr, LDH, and serum albumin, were obtained from biochemical testing data.

### 2.5. Statistical analysis

Currently, there is no consensus regarding the threshold for excluding variables with missing data. In a study by Zhang et al, variables with more than 80% missing values were excluded.^[30]^ In the present study, we set a stricter threshold, excluding variables with more than 20% missing values. Prior to each model fitting, we assumed that the data were missing at random and performed multiple imputation using the “mice” package in RStudio (Posit PBC, Boston). The optimal cutoff value for the MHR was determined using the “maxstat” package based on the maximal statistic, and participants were categorized into low and high MHR groups accordingly.

To explore the potential nonlinear association between MHR and all-cause mortality in stroke patients, we employed restricted cubic spline (RCS) analysis. After testing models with 3 to 7 knots, we selected a 3-knot model based on the lowest Akaike Information Criterion value, and the inflection point was determined by examining the shape of the RCS curve. A weighted Cox proportional hazards model was used to assess the independent association between MHR and all-cause mortality in stroke patients. Three models were constructed: Model 1 (unadjusted), Model 2 (adjusted for age, sex, race, and PIR), and Model 3 (further adjusted for smoking status, hypertension, BMI, cardiovascular disease, urinary albumin, red blood cell count, BUN, Cr, LDH, and albumin).

Survival probabilities across different MHR levels were compared using the Kaplan–Meier method, and group differences were evaluated using the log-rank test. Stratified and interaction analyses were conducted by age, sex, PIR, smoking status, hypertension, and stroke subtype. In addition, mediation analysis was performed to investigate the mediating roles of BMI, waist circumference, white blood cell count, BUN, Cr, and triglycerides in the association between MHR and all-cause mortality in stroke patients. All analyses were performed using R software (version 4.4.2), and a 2-sided *P*-value < .05 was considered statistically significant.^[31]^

## 3. Results

### 3.1. Baseline characteristics of study participants

Based on the inclusion and exclusion criteria, a total of 1513 participants were selected from the NHANES database for this study. Multiple imputation was performed to handle missing values, ensuring that the distribution of variables remained consistent before and after imputation (see Table S1, Supplemental Digital Content, https://links.lww.com/MD/Q374). The optimal cutoff value for the MHR was determined to be 0.47. Accordingly, participants were divided into 2 groups: the high MHR group (MHR > 0.47, n = 631) and the low MHR group (MHR ≤ 0.47, n = 882) (see Table 1 and Figure S1, Supplemental Digital Content, https://links.lww.com/MD/Q373). Compared with participants in the low MHR group, those in the high MHR group exhibited significantly higher levels of urinary albumin, white blood cell count, and triglycerides (see Table 1).

**Table 1:** Baseline characteristics of included participants.

Variable	Overall (N = 1513)^*^	Lower MHR (N = 882)^*^	Higher MHR (N = 631)^*^	*P*-value^†^
Age (yr)	66.57 (13.17)	66.37 (12.90)	66.85 (13.55)	.20
Age group				.40
Old	1129 (74.62%)	651 (73.81%)	478 (75.75%)	
Young	384 (25.38%)	231 (26.19%)	153 (24.25%)	
Gender				<.001
Female	777 (51.35%)	546 (61.90%)	231 (36.61%)	
Male	736 (48.65%)	336 (38.10%)	400 (63.39%)	
Race				<.001
Mexican American	153 (10.11%)	94 (10.66%)	59 (9.35%)	
Non-Hispanic Black	395 (26.11%)	275 (31.18%)	120 (19.02%)	
Non-Hispanic White	781 (51.62%)	413 (46.83%)	368 (58.32%)	
Other Hispanic	82 (5.42%)	43 (4.88%)	39 (6.18%)	
Other race	102 (6.74%)	57 (6.46%)	45 (7.13%)	
PIR				.013
≤1.3	606 (40.05%)	367 (41.61%)	239 (37.88%)	
1.3–3.5	645 (42.63%)	349 (39.57%)	296 (46.91%)	
>3.5	262 (17.32%)	166 (18.82%)	96 (15.21%)	
Smoking history				<.001
Nonsmoker	601 (39.72%)	390 (44.22%)	211 (33.44%)	
Smoker	912 (60.28%)	492 (55.78%)	420 (66.56%)	
BMI	29.70 (6.72)	29.12 (6.74)	30.50 (6.62)	<.001
Weight (kg)	131.71 (805.13)	144.21 (881.95)	114.24 (683.75)	<.001
Waist (cm)	103.74 (15.47)	101.19 (15.33)	107.30 (14.96)	<.001
Diabetes				<.001
No	956 (63.19%)	595 (67.46%)	361 (57.21%)	
Borderline	54 (3.57%)	32 (3.63%)	22 (3.49%)	
Yes	503 (33.25%)	255 (28.91%)	248 (39.30%)	
Hypertension				.014
No	363 (23.99%)	232 (26.30%)	131 (20.76%)	
Yes	1150 (76.01%)	650 (73.70%)	500 (79.24%)	
Cardiovascular diseases				<.001
No	1237 (81.76%)	750 (85.03%)	487 (77.18%)	
Yes	276 (18.24%)	132 (14.97%)	144 (22.82%)	
Albumin urine (µg/mL)	153.33 (666.72)	137.18 (709.69)	175.91 (601.36)	<.001
Red blood cell count (million cells/µL)	4.50 (0.56)	4.45 (0.53)	4.58 (0.60)	<.001
Mean cell hemoglobin (pg)	30.41 (2.73)	30.48 (2.89)	30.31 (2.50)	.53
White blood cell count (1000 cells/µL)	7.42 (2.19)	6.76 (1.97)	8.33 (2.16)	<.001
Hematocrit (%)	37.12 (44.58)	36.50 (42.63)	37.99 (47.20)	.19
Blood urea nitrogen (mg/dL)	17.59 (9.49)	16.62 (8.61)	18.95 (10.45)	<.001
Total protein (g/dL)	7.08 (0.52)	7.06 (0.52)	7.10 (0.51)	.19
Total bilirubin (mg/dL)	0.65 (0.32)	0.66 (0.32)	0.65 (0.32)	.20
Chloride (mmol/L)	103.05 (3.62)	103.14 (3.63)	102.92 (3.61)	.18
Aspartate aminotransferase (AST) (U/L)	24.68 (11.79)	25.15 (13.33)	24.02 (9.19)	.42
Total calcium (mg/dL)	9.39 (0.45)	9.41 (0.46)	9.38 (0.43)	.22
Creatinine refrigerated serum (mg/dL)	1.16 (0.82)	1.08 (0.74)	1.26 (0.92)	<.001
Alanine aminotransferase (ALT) (U/L)	21.99 (13.71)	21.74 (14.52)	22.34 (12.50)	.033
Cholesterol refrigerated serum (mg/dL)	185.04 (47.31)	192.80 (48.00)	174.19 (44.14)	<.001
Lactate dehydrogenase (LDH) (U/L)	142.97 (35.81)	144.66 (37.02)	140.61 (33.92)	.050
Albumin refrigerated serum (g/dL)	4.07 (0.36)	4.08 (0.35)	4.05 (0.38)	.070
Potassium (mmol/L)	4.08 (0.44)	4.05 (0.45)	4.11 (0.44)	.003
Sodium (mmol/L)	139.54 (2.91)	139.67 (2.96)	139.36 (2.84)	.019
Triglycerides refrig serum (mg/dL)	155.12 (93.48)	132.87 (75.91)	186.21 (106.08)	<.001

BMI = body mass index, MHR = monocyte-to-high-density lipoprotein cholesterol ratio, PIR = ratio of family income to poverty.

Continuous variables are presented as the mean and 95% confidence interval.

Category variables are described as the percentage and 95% confidence interval.

* Mean (SD) or frequency (%).

† Wilcoxon rank sum test; Pearson Chi-squared test.

### 3.2. Associations between MHR and all-cause mortality in patients with stroke

#### 3.2.1. RCS analysis

**Figure 2.**
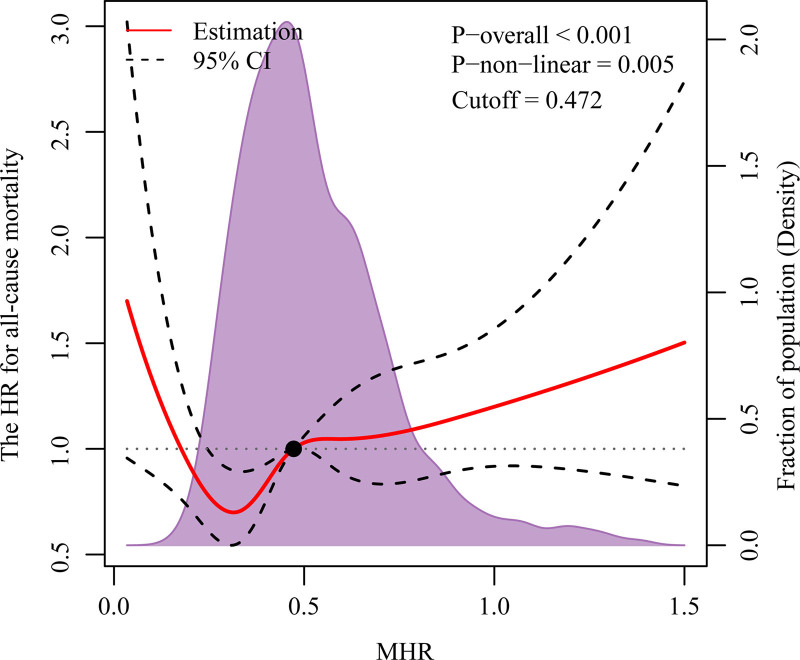
Association between MHR in all-cause mortality patients visualized by restricted cubic spline. MHR = monocyte-to-high-density lipoprotein cholesterol ratio.

The RCS analysis demonstrated that the MHR was positively associated with all-cause mortality in stroke patients. Specifically, as MHR increased, the all-cause mortality risk among stroke patients rose significantly. The analysis revealed a significant overall trend in the association between MHR and all-cause mortality risk (*P* for overall trend < .001), with a notably significant nonlinear relationship (*P* for nonlinearity = .005), as illustrated in Figure 2.

#### 3.2.2. Weighted Cox regression analysis

The weighted Cox regression analysis also demonstrated a positive association between the MHR and all-cause mortality in stroke patients. In the unadjusted Model 1, each unit increase in MHR as a continuous variable was associated with a significantly elevated risk of all-cause mortality (HR = 1.905, 95% confidence interval [CI]: 1.38–2.631, *P* < .001). This association remained marginally significant after multivariable adjustments in Model 2 (HR = 1.389, 95% CI: 0.977–1.976, *P* = .0674) and Model 3 (HR = 1.369, 95% CI: 0.957–1.957, *P* = .0851).

Furthermore, in Model 1, when MHR was analyzed categorically as lower (≤0.47) and higher (>0.47) groups, patients in the higher MHR group exhibited a significantly increased risk of all-cause mortality compared to the lower MHR group (HR = 1.269, 95% CI: 1.133–1.42, *P* < .001). A consistent trend was observed in multivariable-adjusted models: Model 2 (HR = 1.164, 95% CI: 1.035–1.308, *P* = .011) and Model 3 (HR = 1.172, 95% CI: 1.04–1.32, *P* = .009), as presented in Table 2.

**Table 2:** The relationships between MHR and In-hospital mortality in all-cause death.

		Model 1			Model 2			Model 3		
Characterisitic	Case (%)	HR	95% CI	*P*-value	HR	95% CI	*P*-value	HR	95% CI	*P*-value
MHR										
MHR continuous		1.905	(1.38–2.631)	*P* < .001	1.389	(0.977–1.976)	.0674	1.369	(0.957–1.957)	.0851
MHR category										
Lower MHR	58.29	Ref			Ref			Ref		
Higher MHR	41.71	1.269	(1.133–1.42)	*P* < .001	1.164	(1.035–1.308)	.011	1.172	(1.04–1.32)	.009

BMI = body mass index, CI = confidence interval, HR = hazard ratio, MHR = monocyte-to-high-density lipoprotein cholesterol ratio.

The Model 1 was non-adjusted.

The Model 2 was adjusted by age years, gender.

The Model 3 was adjusted by age years, gender, smoking history, BMI, albumin urine, total protein.

The stratified Cox regression analysis evaluating the association between the MHR and all-cause mortality in stroke patients revealed distinct patterns across MHR subgroups. In the lower MHR group (HR = 0.836, 95% CI: 0.618–1.132, *P* = .2467), the risk of mortality showed a nonsignificant protective trend. Conversely, the higher MHR group exhibited a significantly increased risk of all-cause mortality (HR = 1.236, 95% CI: 1.094–1.396, *P* < .0001). The log-likelihood ratio test indicated significant overall model fit (*P* < .0001).

These results indicate that although the hazard ratio (HR) in the lower MHR group was <1, the CI crossed unity and statistical significance was not achieved (*P* = .2467), suggesting only a nonsignificant trend. In contrast, a higher MHR was significantly associated with increased all-cause mortality in stroke patients and was identified as an independent risk factor (*P* < .0001), as detailed in Table S2, Supplemental Digital Content, https://links.lww.com/MD/Q374.

#### 3.2.3. Survival curve analysis

**Figure 3.**
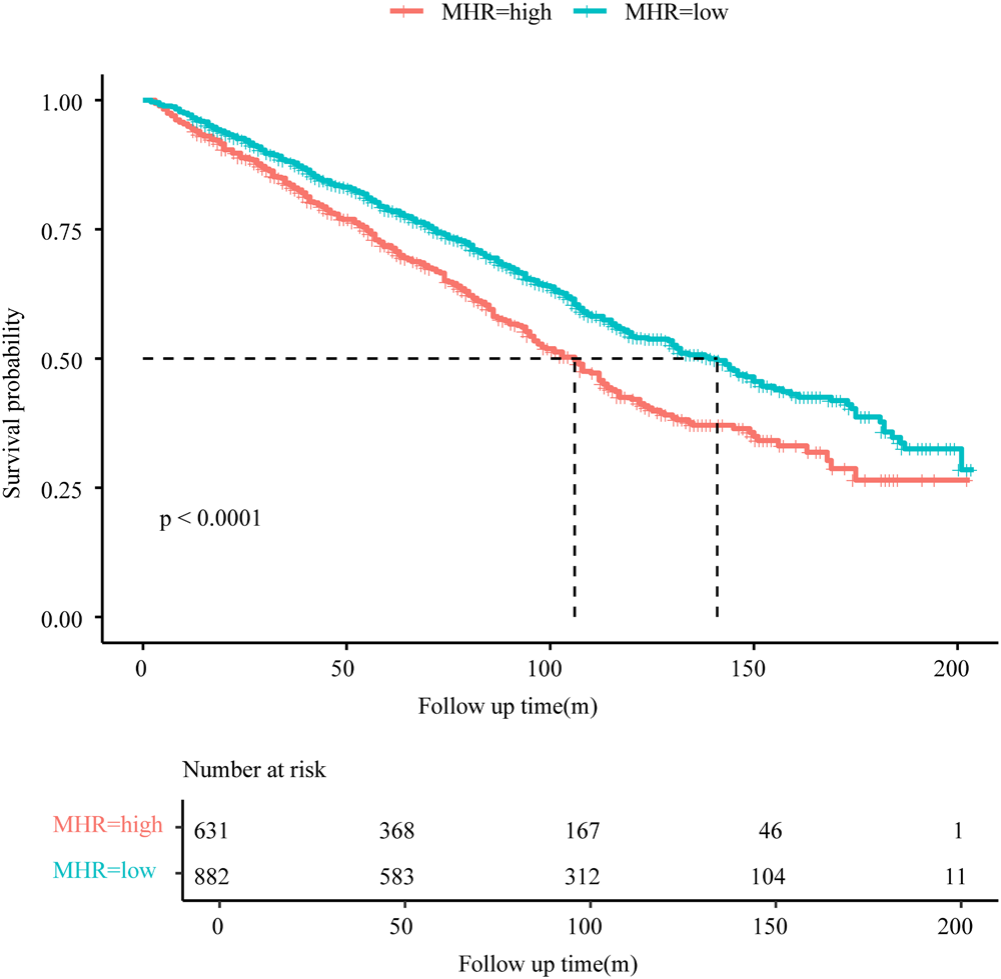
Kaplan–Meier curves of the survival rate with higher (>0.47) and lower (≤0.47) MHR values. MHR = monocyte-to-high-density lipoprotein cholesterol ratio.

The Kaplan–Meier survival curves demonstrated a significant divergence in all-cause mortality between MHR subgroups in stroke patients. The higher MHR group exhibited a lower initial survival probability at the onset of follow-up, with a steeper decline in survival over time, indicating an elevated risk of early mortality. In contrast, the lower MHR group maintained a higher survival probability, and its survival curve declined more gradually, suggesting better survival outcomes during the follow-up period. The log-rank test confirmed a statistically significant difference in survival between the higher and lower MHR groups (*P* < .0001), as illustrated in Figure 3.

Consistent with the Cox regression findings, these results reinforce that elevated MHR levels are strongly associated with adverse survival outcomes in stroke patients.

#### 3.2.4. Subgroup analysis

Subgroup analyses stratified by age, sex, race, PIR, smoking history, and hypertension status were performed to evaluate the association between the MHR and all-cause mortality in stroke patients. Across all subgroups, the higher MHR group consistently demonstrated an elevated mortality risk compared to the lower MHR group. Notably, no significant interaction effects were observed between MHR and these stratification variables (*P* for interaction > .05 for all), indicating the robustness of the MHR-mortality association irrespective of baseline characteristics, as detailed in Table 3.

**Table 3:** Subgroup analysis of the associations between MHR and in-hospital mortality among all-cause death.

Characteristics	Lower MHR	Higher MHR		*P* interaction
		HR (95% CI)	*P*-value	
Age group				.194
Old	Ref	1.31 (1.11–1.54)	.002	
Young	Ref	1.94 (1.1–3.41)	.021	
Gender				.925
Female	Ref	1.24 (0.95–1.6)	.109	
Male	Ref	1.24 (1–1.53)	.047	
Race				.637
Mexican American	Ref	1.34 (0.79–2.27)	.282	
Non-Hispanic Black	Ref	1.24 (0.85–1.8)	.268	
Non-Hispanic White	Ref	1.37 (1.12–1.67)	.002	
Other Hispanic	Ref	0.68 (0.26–1.76)	.43	
Other race	Ref	1.69 (0.73–3.9)	.222	
PIR				.701
≤1.3	Ref	1.45 (1.12–1.89)	.005	
1.3–3.5	Ref	1.33 (1.05–1.67)	.016	
3.5	Ref	1.16 (0.77–1.75)	.482	
Smoking history				.711
Nonsmoker	Ref	1.29 (0.98–1.69)	.065	
Smoker	Ref	1.39 (1.14–1.69)	.001	
Diabetes				.511
No	Ref	1.29 (1.05–1.59)	.016	
Borderline	Ref	0.86 (0.37–2)	.721	
Yes	Ref	1.5 (1.15–1.97)	.003	
Hypertension				.203
No	Ref	1.62 (1.14–2.3)	.007	
Yes	Ref	1.29 (1.08–1.54)	.005	

CI = confidence interval, MHR = monocyte-to-high-density lipoprotein cholesterol ratio, PIR = ratio of family income to poverty.

#### 3.2.5. Mediation analysis of MHR and all-cause mortality in stroke patients

**Figure 4.**
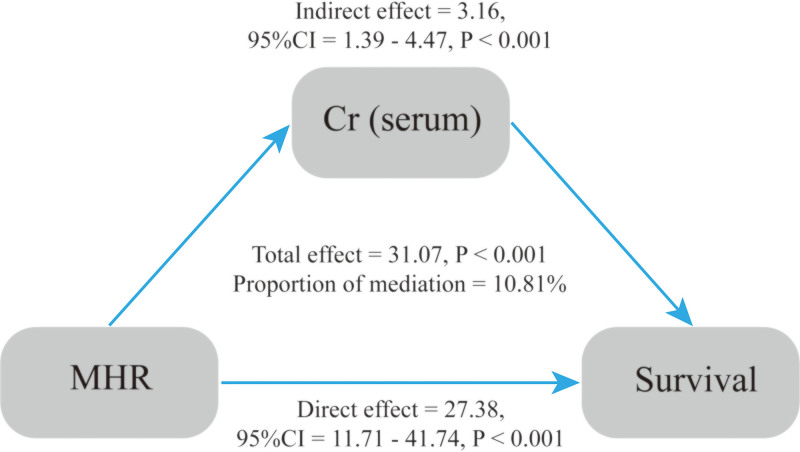
The mediating effect between MHR and survival. MHR = monocyte-to-high-density lipoprotein cholesterol ratio.

This study examined the mediating roles of BMI, waist circumference, white blood cell count, BUN, Cr, and triglycerides in the association between the MHR and all-cause mortality in stroke patients. Among these variables, Cr mediated 10.81% of the total association between MHR and mortality. The total effect of MHR on mortality was 31.07 (*P* < .001), comprising a direct effect of 27.38 (*P* < .001) and an indirect effect mediated through Cr of 3.16 (*P* < .001), as shown in Tables S3 and S4, Supplemental Digital Content, https://links.lww.com/MD/Q374 and Figure 4.

## 4. Discussion

This study is the first to investigate the association between the MHR and all-cause mortality in patients with stroke using data from the NHANES. Based on health examination data from 1513 stroke participants in the NHANES database, we found a positive association between MHR and all-cause mortality in patients with stroke. Mediation analysis further revealed that Cr played a mediating role in the relationship between MHR and mortality. These findings remained robust across various sensitivity and stratified analyses.

Notably, several studies based on the NHANES database have identified MHR as a potential biomarker for a range of clinical conditions. For example, a large-scale study involving 42,178 participants demonstrated that MHR was closely associated with the prevalence of cardiorenal syndrome in the U.S. population and may serve as a useful predictive tool for this condition.^[32]^ Jiang et al reported a significant association between MHR and cardiovascular mortality in the U.S. population.^[33]^ Similarly, Wang et al observed a positive relationship between MHR and the risk of nephrolithiasis among American adults using NHANES data,^[34]^ while Liu et al found that elevated MHR levels were positively correlated with gallstone prevalence in the same population.^[35]^

However, existing NHANES-based studies have primarily focused on the association between MHR and various metabolic or cardiovascular diseases. To date, no studies have specifically explored the relationship between MHR and stroke in the U.S. population. Interestingly, stroke shares several pathophysiological and prognostic features with cardiovascular and metabolic diseases, particularly in the domains of chronic inflammation, endothelial dysfunction, and oxidative stress.^[36–39]^ These shared mechanisms contribute not only to disease initiation but also to long-term adverse outcomes, including mortality. For instance, systemic inflammation and monocyte activation are recognized predictors of poor prognosis in both coronary artery disease and ischemic stroke,^[40–42]^ while reduced HDL-C levels have similarly been associated with worse outcomes across these conditions.^[43,44]^ Given that MHR integrates both pro-inflammatory (monocytes) and anti-inflammatory/antioxidative (HDL-C) components, it serves as a biologically plausible and clinically relevant prognostic marker in diseases driven by these common pathways. Therefore, the established utility of MHR in predicting outcomes in cardiovascular and metabolic disorders further supports its potential value in risk stratification among stroke patients. Therefore, our study aimed to address this gap by examining the potential association between MHR and all-cause mortality in patients with stroke using NHANES data from the 1999 to 2018 and 2021 to 2023 cycles. A total of 1513 participants were ultimately included in the study from an initial pool of 113,249 individuals. Our results revealed a positive linear relationship between MHR levels and all-cause mortality in patients with stroke.

In support of our findings, several recent studies based on non-U.S. populations have also highlighted the clinical relevance of MHR in stroke-related outcomes. For instance, Xu et al, using data from the Third China National Stroke Registry, reported that elevated MHR independently predicted all-cause mortality and poor functional outcomes in patients with ischemic stroke.^[45]^ Li et al found that MHR might be a useful biomarker for predicting post-stroke depression.^[46]^ In a large Chinese community-based cohort, Wang et al observed a positive association between MHR levels and the incidence of ischemic stroke.^[47]^ Similarly, Liu et al, utilizing data from the Henan Stroke Registry of the First Affiliated Hospital of Zhengzhou University, demonstrated that MHR was an independent predictor of poor prognosis in patients with acute ischemic stroke.^[48]^ Bolayir et al reported that MHR may be a risk factor for predicting 30-day mortality in patients with acute ischemic stroke,^[49]^ and Xia et al further found that elevated MHR levels were associated with an increased risk of hemorrhagic transformation in patients receiving intravenous thrombolysis for acute ischemic stroke.^[50]^ Collectively, these findings from various databases corroborate the results of our current study and further support the potential value of MHR as a predictive marker of stroke.

MHR reflects the balance between pro-inflammatory monocytes and the anti-inflammatory, antioxidative, and vasculoprotective properties of HDL. Elevated monocyte counts can promote atherogenesis, destabilization of vulnerable plaques, microvascular injury, and post-stroke inflammatory cascades, leading to worse neurological recovery and increased mortality.^[51,52]^ Conversely, reduced HDL levels or impaired HDL functionality diminish reverse cholesterol transport, antioxidant capacity, and endothelial protection, further exacerbating vascular injury and thromboinflammatory responses.^[53–55]^ A higher MHR thus represents a dual burden of enhanced inflammatory activity and diminished vascular protection, which may contribute to larger infarct size, blood–brain barrier dysfunction, oxidative stress, and secondary neurovascular damage. These pathophysiological links provide a plausible explanation for the observed association between elevated MHR and increased all-cause mortality in stroke patients.

Serum creatinine serves as a readily obtainable surrogate of kidney function, and renal impairment is consistently associated with adverse post-stroke outcomes, including higher all-cause mortality.^[56,57]^ Mechanistically, reduced renal function promotes a pro-inflammatory and pro-oxidative milieu, endothelial dysfunction, and pro-thrombotic tendencies.^[58–60]^ In parallel, both the quantity and functionality of HDL can be compromised in chronic kidney disease, including alterations in cholesterol efflux capacity, antioxidant/anti-inflammatory properties, and protein cargo.^[61–63]^ These changes may attenuate HDL’s protective effects while favoring monocyte activation and atherothrombotic risk, thereby strengthening the prognostic signal captured by MHR.

Consistent with these pathways, our mediation analysis indicated that Cr partially mediated the relationship between MHR and all-cause mortality (proportion mediated: 10.81%). This suggests that renal function, as indicated by Cr, may be an important biological link connecting systemic inflammation and lipid metabolism to post-stroke outcomes. Elevated MHR reflects an imbalance between pro-inflammatory monocytes and anti-inflammatory HDL-C, contributing to vascular injury and atherosclerosis, which are central to stroke prognosis.^[33,64,65]^ At the same time, impaired renal function is known to aggravate systemic inflammation and oxidative stress, which can amplify cerebrovascular damage. Accumulating evidence suggests that impaired renal function is an independent and significant predictor of mortality in stroke patients, further supporting its relevance in post-stroke risk assessment.^[66–68]^ Thus, Cr may serve as a surrogate indicator of this pathological interplay.

These findings emphasize the interdependence between inflammation, lipid homeostasis, and renal function in shaping stroke prognosis. Creatinine, as a widely accessible marker of renal status, could complement MHR in improving risk stratification among stroke survivors. The partial mediation effect also indicates that other unmeasured pathways, such as endothelial dysfunction or immune dysregulation, may contribute to the observed association and should be investigated in future studies.

## 5. Strengths and limitations

This study possesses several important strengths that enhance the credibility and impact of its findings. Firstly, this study is the first to investigate the association between the MHR and all-cause mortality in patients with stroke using data from the NHANES. Secondly, the utilization of NHANES data, which is based on a sophisticated multistage, probability-based sampling framework, allows the findings to reflect the characteristics of the general U.S. population. This strengthens the external validity and practical relevance of the study results. Thirdly, the study has taken into account potential confounding factors, allowing for a more accurate assessment of the association between MHR levels and all-cause mortality. Notably, our study is the first to reveal the potential mediating role of Cr in the relationship between MHR and mortality in patients with stroke.

However, this study has several limitations. First, the findings of this study are based on data from stroke patients in the United States; therefore, additional research is crucial to evaluate their applicability to stroke populations in other countries. Implementing large-scale studies across broader geographic regions and more diverse populations would further improve the external validity and generalizability of the conclusions. Secondly, NHANES data are based on self-reported questionnaires, which may introduce recall bias. Third, although we adjusted for a wide range of confounding variables, residual confounding cannot be entirely ruled out, and unmeasured factors may still influence the observed association between MHR and mortality. Overall, although our study offers important insights, the limitations outlined should be carefully considered when drawing conclusions from the findings.

## 6. Conclusion

Our findings demonstrate a significant and independent association between elevated MHR and the risk of all-cause mortality among stroke patients in the United States. Notably, Cr was identified as a potential mediator in this relationship. These results suggest that MHR may serve as a simple and practical biomarker for identifying high-risk individuals and informing targeted intervention strategies. Further prospective studies are warranted to confirm these findings and to elucidate the underlying mechanisms.

## Author contributions

**Conceptualization:** Jia Ouyang, Bin Wang.

**Data curation:** Bo Hei, Jia Ouyang, Jixia Fang, Qun Gao, Jingru Zhou.

**Formal analysis:** Bo Hei, Jixia Fang, Bin Wang.

**Funding acquisition:** Jia Ouyang.

**Investigation:** Qun Gao, Bin Wang, Jingru Zhou.

**Methodology:** Jixia Fang, Qun Gao, Bin Wang.

**Project administration:** Bo Hei.

**Resources:** Jixia Fang.

**Software:** Jixia Fang, Bin Wang.

**Supervision:** Jia Ouyang, Jixia Fang, Bin Wang, Jingru Zhou.

**Validation:** Jingru Zhou.

**Writing – original draft:** Bo Hei, Jia Ouyang, Jixia Fang.

**Writing – review & editing:** Qun Gao, Bin Wang, Jingru Zhou.

## Supplementary Material




