# Supplementary figures and images for: Association between monocyte-to-high-density lipoprotein cholesterol ratio and all-cause mortality in stroke patients: Exploring the potential mediating role of serum creatinine in a NHANES-based study
Source: Medicine (Baltimore). 2025 Oct 17;104(42):e45298. doi: 10.1097/MD.0000000000045298 (PMC12537246; doi:10.1097/MD.0000000000045298)

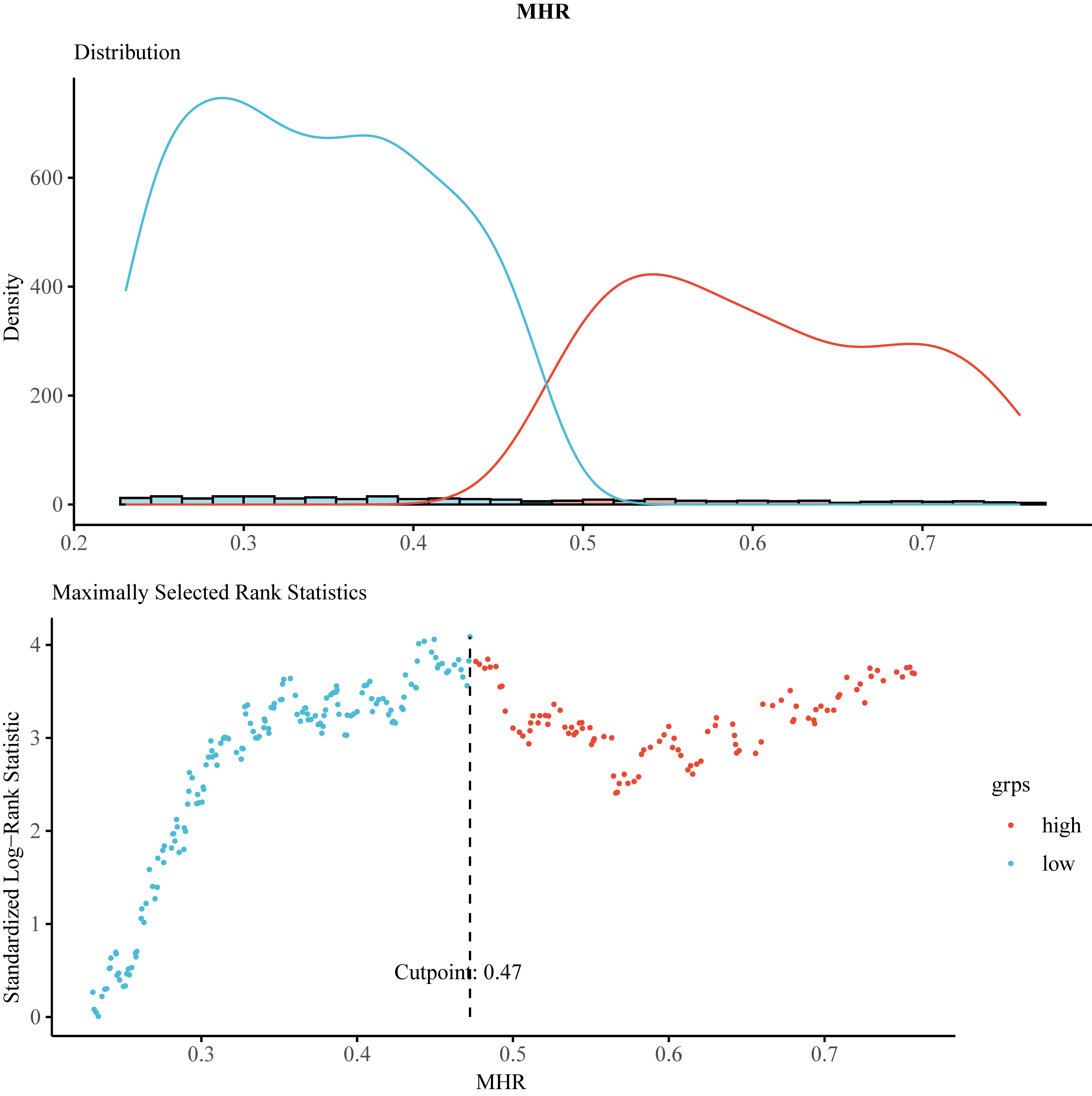

Supplement: Supplementary file 2 [file medi-104-e45298-s002.tif]
